# Genomic characterization of cocirculating *Corynebacterium diphtheriae* and non-diphtheritic *Corynebacterium* species among forcibly displaced Myanmar nationals, 2017–2019

**DOI:** 10.1099/mgen.0.001085

**Published:** 2023-09-15

**Authors:** Lingzi Xiaoli, Yanhui Peng, Margaret M. Williams, Marlon Lawrence, Pamela K. Cassiday, Janessa S. Aneke, Lucia C. Pawloski, Sadhona Rani Shil, Mamun Or Rashid, Proshanta Bhowmik, Lauren M. Weil, Anna M. Acosta, Tahmina Shirin, Zakir Hossain Habib, M. Lucia Tondella, Michael R. Weigand

**Affiliations:** ^1^​ ASRT, Inc, Atlanta, GA, USA; ^2^​ Division of Bacterial Diseases, National Center for Immunization and Respiratory Diseases, Centers for Disease Control and Prevention, Atlanta, GA, USA; ^3^​ Laboratory Leadership Service, Division of Scientific Education and Professional Development, Center for Surveillance, Epidemiology, and Laboratory Services, Centers for Disease Control and Prevention, Atlanta, GA, USA; ^4^​ IHRC, Inc., Atlanta, GA, USA; ^5^​ Institute of Epidemiology, Disease Control & Research, National Influenza Center, Dhaka, Bangladesh; ^6^​ Epidemic Intelligence Service, Division of Scientific Education and Professional Development, Center for Surveillance, Epidemiology, and Laboratory Services, Centers for Disease Control and Prevention, Atlanta, GA, USA; ^†^​ Present address: Division of Foodborne, Waterborne, and Environmental Diseases, National Center for Emerging and Zoonotic Infectious Diseases, Centers for Disease Control and Prevention, Atlanta, GA, USA; ^‡^​ Present address: Division of Healthcare Quality Promotion, National Center for Emerging and Zoonotic Infectious Diseases, Centers for Disease Control and Prevention, Atlanta, GA, USA; ^§^​ Present address: Public Health Laboratory, Virgin Islands Department of Health, US Virgin Islands, USA; ^#^​ Present address: Université de Paris Cité, Learning Planet Institute, Paris, France; ^¶^​ Present address: Division of Healthcare Quality Promotion, National Center for Emerging and Zoonotic Infectious Diseases, Centers for Disease Control and Prevention, Atlanta, GA, USA; ^**^​ Present address: Director of Medical and Clinical Affairs, GSK Vaccines, USA

**Keywords:** *Corynebacterium*, diphtheria, genomic epidemiology, outbreak, whole-genome sequencing

## Abstract

Respiratory diphtheria is a serious infection caused by toxigenic *Corynebacterium diphtheriae *, and disease transmission mainly occurs through respiratory droplets. Between 2017 and 2019, a large diphtheria outbreak among forcibly displaced Myanmar nationals densely settled in Bangladesh was investigated. Here we utilized whole-genome sequencing (WGS) to characterize recovered isolates of *C. diphtheriae* and two co-circulating non-diphtheritic *Corynebacterium* (NDC) species – *C. pseudodiphtheriticum* and *C. propinquum. C. diphtheriae* isolates recovered from all 53 positive cases in this study were identified as toxigenic biovar mitis, exhibiting intermediate resistance to penicillin, and formed four phylogenetic clusters circulating among multiple refugee camps. Additional sequenced isolates collected from two patients showed co-colonization with non-toxigenic *C. diphtheriae* biovar gravis, one of which exhibited decreased susceptibility to the first-line antibiotics and harboured a novel 23-kb multidrug resistance plasmid. Results of phylogenetic reconstruction and virulence-related gene contents of the recovered NDC isolates indicated they were likely commensal organisms, though 80.4 %(45/56) were not susceptible to erythromycin, and most showed high minimum inhibition concentrations against azithromycin. These results demonstrate the high resolution with which WGS can aid molecular investigation of diphtheria outbreaks, through the quantification of bacterial genetic relatedness, as well as the detection of virulence factors and antibiotic resistance markers among case isolates.

## Data Summary

Raw and assembled genome sequence data have been deposited in the NCBI Sequence Read Archive and GenBank, respectively, organized under BioProject accession number PRJNA541851. The complete genome assemblies for eight *C. diphtheriae* isolates are available under accession numbers CP040519–CP040523, CP040525, CP040526, and CP091095.

**Impact Statement**

Thanks to the widespread application of effective vaccines, respiratory diphtheria cases have decreased significantly worldwide. However, large outbreaks still occur in regions with limited healthcare infrastructure or inadequate vaccination coverage. The present work supplements microbiological characterization with whole-genome sequencing to provide added depth for understanding strains of *C. diphtheriae* circulating in one such outbreak among forcibly displaced Myanmar nationals densely settled in Bangladesh. Our evaluation revealed that disease in this outbreak resulted from concurrent transmission of multiple distinct toxigenic strains, distinguishable by discrete genotypes. While these genomic data were not available in real-time to inform immediate public health action, they may reinforce ongoing and future public health interventions. Our results confirm that toxigenic *C. diphtheriae* strains circulate regionally and routine diphtheria toxoid vaccination, as well as continued surveillance and antimicrobial resistance testing, are critical for the response to future regional outbreaks.

## Introduction

Diphtheria, caused by toxigenic strains of the Gram-positive bacterium *Corynebacterium diphtheriae *, can lead to upper respiratory tract disease with symptoms of sore throat, mild fever and gray-white pseudomembrane on the tonsils, larynx or pharynx. Transmission occurs through respiratory droplets, and the overall case-fatality rate is 5–10 % in unvaccinated and untreated individuals, but can vary widely in resource-limited settings where case fatality rates of up to 30 % have been documented [1]. Current treatments include diphtheria antitoxin (DAT) and antibiotics (penicillin and erythromycin) [2–4]. Following widespread application of effective vaccines against the diphtheria toxin, respiratory diphtheria cases have decreased significantly worldwide. However, large diphtheria outbreaks have been increasingly reported in regions experiencing disruptions to healthcare infrastructure or inadequate vaccination coverage, often the result of political conflict [5–7]. Multiple epidemiological investigations of one such outbreak during 2017–2019 among forcibly displaced Myanmar nationals settled in refugee camps in Bangladesh have been reported recently [8, 9].

Molecular tools, such as multi-locus sequencing typing (MLST), have been used to support diphtheria outbreak investigations by elucidating transmission patterns or the spread of epidemic *C. diphtheriae* genotypes [10–15]. More recently, genomic approaches, including core-genome MLST (cgMLST) and genome-wide single nucleotide polymorphisms (SNPs), provide a higher discriminatory power to accurately quantify bacterial genetic relatedness among case isolates as well as detect antibiotic resistance and virulence gene determinants [7, 16–24]. Previous studies leveraging whole-genome sequencing (WGS) to investigate outbreaks have suggested that isolates within transmission networks typically differ by <150 SNPs and isolates from epidemiologically linked cases may differ by <40 SNPs, enabling cluster delineation via simple quantification of bacterial genetic distance [16, 17, 19, 22, 24]. Furthermore, epidemiological or geographical associations among isolates can be predicted using similar thresholds, because the *C. diphtheriae* population exhibits sufficient sequence diversity that sporadic isolates often differ by >10 000 SNPs [16].

The genus *Corynebacterium* includes other species capable of respiratory infection in humans, animals or both [25], including the zoonotic species *C. ulcerans* and, rarely, *C. pseudotuberculosis* that may also produce diphtheria toxin [26–29]. However, most bacteria in this genus do not produce toxin and are classified as the non-diphtheritic *Corynebacterium* (NDC) species [25, 30]. NDCs are considered commensal members of the healthy microflora but have gradually been recognized as emerging opportunistic pathogens capable of causing endocarditis, pneumonitis, bronchiectasis or skin infections [31–36]. Most notably, *C. pseudodiphtheriticum* can cause exudative pharyngitis resembling the typical toxin-mediated pseudomembrane associated with *C. diphtheriae* [32, 37–40], leading to clinical and laboratory diagnostic challenges, particularly during a diphtheria outbreak investigation [8]. Few genomic studies of NDC isolates have been reported and the ecology of these human-associated species, including their potential for causing disease, remains poorly understood [41, 42].

Our previous epidemiologic and laboratory investigation recovered both *C. diphtheriae* and NDC isolates during a 7 day period of a large diphtheria outbreak among forcibly displaced Myanmar nationals settled in Bangladesh [8]. In this study, we utilized microbiological, WGS and bioinformatics tools to further characterize the molecular dynamics of this diphtheria outbreak, including the co-circulation of NDC species*,* with isolates recovered during an 18 week period of the investigation. The results provide detailed profiles of the poly-clonal spread of toxigenic *C. diphtheriae* among vulnerable people, as well as unique genomic evidence of the presumably commensal distribution of NDC species within the same population. Together these data demonstrate the informative contribution of WGS to investigations of diphtheria outbreaks and co-circulating NDC species.

## Methods

### Isolate sampling

In total, 56 *C *. *diphtheriae* isolates were recovered from 53 positive diphtheria cases within the Bangladesh outbreak and sent to CDC for further genomic analyses. Notably, 31/53(58 %) of cases were sampled during the 7 day intensive study period of 19–25 December 2017, as previously reported [8], including three patients for which one isolate each was recovered from nasal and throat swabs. The remaining 22 cases, one sampled before this study period and 21 sampled after, each yielded only one *C. diphtheriae* isolate. Additionally, 56 NDC isolates were randomly collected from nasal swabs of one diphtheria positive and 55 diphtheria negative patients during the entire 18 week study course (9 December 2017 to 7 April 2018). The camp and collection date for each isolate are indicated in Table S1, available in the online version of this article.

## Microbiological characterization

All isolates were grown on trypticase soy agar with 5 % sheep blood (BBL, Sparks, MD) and biochemically identified to biovar using API Coryne Strips (bioMérieux, Durham, NC). Toxin production was confirmed by Elek test, and minimum inhibition concentration (MIC) of 11 different antibiotics (amoxicillin, azithromycin, clarithromycin, clindamycin, daptomycin, erythromycin, levofloxacin, meropenem, penicillin, rifampin and vancomycin) was determined by Etest (bioMérieux, Durham, NC). The Clinical and Laboratory Standard Institute (CLSI) 2015 guidelines have been used to interpretate the antibiotic resistance categories: erythromycin: ‘sensitive’ (MIC<=0.5 µg ml^−1^), ‘intermediate’ (MIC=1 µg ml^−1^) or resistant (MIC>=2 µg ml^−1^); penicillin: ‘sensitive’ (MIC<=0.12 µg ml^−1^), ‘intermediate’ (MIC >0.25–2.0 µg ml^−1^) or resistant (MIC>=4.0 µg ml^−1^). As clinical breakpoints for *Corynebacterium* species are not defined for amoxicillin, azithromycin, clarithromycin or levofloxacin, these were not assigned an interpretive category. A full list of interpretive categories is provided in Table S1.

### Whole-genome sequencing and assembly

Isolates were grown on trypticase soy agar with 5 % sheep blood at 37 °C for 24 h. Genomic DNA was extracted using the Maxwell RSC Whole Blood DNA Kit (Promega, San Luis Obispo, CA) and concentrations were determined using the Qubit dsDNA Broad Range Quantification Kit (Thermo Fisher Scientific, Waltham, MA). Illumina paired-end libraries were prepared with the NEBNext Ultra DNA Library Prep Kit (New England Biolabs, Ipswich, MA) and sequenced on a MiSeq using v2 Kit (Illumina, San Diego, CA). The Illumina raw reads were checked for quality using FastQC v0.11.5 [43], and then trimmed and filtered with Cutadapt v2.3 [44]. Trimmed reads were *de novo* assembled using SPAdes v3.15.3 [45] and evaluated by quast v4.5 [46].

The genomes of eight *C. diphtheriae* isolates were further subjected to additional long-read sequencing on the PacBio platform (Pacific Biosciences, Menlo Park, CA), selected to represent the observed diversity of MLST sequence types (STs), antibiotic resistance and API profiles. Prior to library preparation, genomic DNA was further purified by salt-chloroform washing [47]. PacBio sequencing libraries were prepared using the SMRTbell Template Prep Kit 1.0 and sequenced on a RSII instrument using Polymerase Binding Kit P6 V2 (Pacific Biosciences, Menlo Park, CA). The *de novo* assembly was performed using the Hierarchical Genome Assembly Process (HGAP) v3 (Pacific Biosciences, Menlo Park, CA). The resulting consensus sequence was manually checked for circularity using Gepard v1.30 [48] and further polished by mapping Illumina reads in CLC Genomics Workbench v20 (Qiagen Digital Insight, Aarhaus, Denmark). The complete genome assemblies were annotated using the NCBI Prokaryotic Genome Annotation Pipeline (PGAP) [49]. Accession numbers for raw and assembled genomic data are listed in Table S1.

### MLST, diphtheria toxin and antibiotic resistance gene detection

MLST allele profiles were determined from the trimmed sequencing reads using srst2 v0.2.0 [50] and stringmlst v0.6.3 [51]. Presence of the diphtheria toxin gene was determined from trimmed reads through srst2 using a subset of virulence genes associated with genus *Corynebacterium* in the virulence factor database (VFDB) as the reference. Further sequence analysis of the diphtheria toxin gene and its promoter region in non-toxigenic toxin gene bearing (NTTB) isolate PC0734 was conducted through alignment using blastn v2.2.26 [52] and read mapping with bowtie2 v2.3.5.1 [53], and compared to the reference strain NCTC13129 (accession no: NC_002935).

Screening for known antibiotic resistance determinants (e.g. macrolide resistance determinant, *ermX*) was performed with srst2 using ARGannot_r3.fasta as the reference for both *C. diphtheriae* and NDC genomes. The genetic context of *ermX* was further analysed in each NDC genome by querying the *ermX*-containing contig from *de novo* assembly in ISfinder [54]. Moreover, 10 kb of flanking sequence upstream and downstream of *ermX* in four complete NDC genomes [42] were compared with blastn v2.2.26 and visualized by Easyfig [55]. In addition, the presence of penicillin-binding proteins (PBP) in *C. diphtheriae* was further assessed by blastx v2.2.26 [52] query of assembled contigs for each of the 56 *C *. *diphtheriae* isolates against eight PBP sequences reported previously [21].

### Whole-genome SNP phylogeny and characterization

Publicly available genomic data for *C. diphtheriae* isolates retrieved from NCBI were comprised of 860 short-read sets (downloaded on 4 February 2021) as well as 29 complete genomes and seven assemblies (downloaded on 4 January 2022). Short-read data were further filtered to include only those with read lengths above 37 bp, average coverage depth of 30–1000×, and confirmed species identification in a linked BioSample record. Therefore, 738 publicly available genomes [NCBI’s Sequence Read Archive (SRA): 704, complete genomes: 27, assemblies: 7] were included with our 56 *C *. *diphtheriae* isolates for building the global phylogenetic tree (*N*_total_=794). Raw reads were trimmed and filtered as described earlier. SNPs were determined by mapping trimmed reads to the reference genome of NCTC13129 (accession no: NC_002935) using Snippy v4.3.8 [56] with default settings. The resulting core SNP alignment was used to estimate the phylogeny using maximum-likelihood with RaxML v8.2.9 [57]. The final tree was visualized with iToL v4 [58]. Construction of subtrees for each cluster followed a similar procedure using a reference genome (listed in Table S1) selected from that cluster. The within-cluster SNP distances were determined relative to the reference’s complete genome using Snippy, and pairwise SNP distances were calculated from the subtree core alignments using SNP-Dist v4.0 [59].

Phylogenies of NDC species were reconstructed in a similar manner using the complete genome assembly of PC1113 (accession no. CP091865) as the reference. In addition, nine NCBI available genomes for *C. propinquum* (*n*=2) and *C. pseudodiphtheriticum* (*n*=7) have been included. Similarly, the pairwise SNP distance for each branch was calculated using SNP-Dist v4.0. Average nucleotide identity (ANI) was calculated against three references (* C. propinquum* accession no. CP068160 and CP068161, *C. pseudodiphtheriticum* accession no. CP091863) using MUMmer V4.0 [60].

### *In silico* plasmid identification

PacBio sequencing (described above) was used to determine the complete plasmid sequence detected in PC0697, designated pSRPD (small resistance plasmid of *C. diphtheriae *). The closed plasmid assembly was annotated using PGAP. The presence of *pbp*-containing unit (PCU) was determined through blastx against the sequence of PCU in plasmid pLRPD (NCBI accession number: CADDFR010000041.1) [21]. The identification of insertion sequence (IS) elements within the PCU was furthered confirmed through IS finder [54]. The presence of macrolide resistance gene (*ermX*) was determined by blastn v2.2.26 against *ermX* in the plasmid of pNG2 (NCBI accession number: WP_011117480.1). blast Ring Image Generator (BRIG) [61] was used to visualize pSRPD’s GC content, GC skew, annotated coding DNA sequences (CDSs), as well as the comparative analysis against pLRPD [21]. Evidence of potential cross-species transfer of pSRPD between *C. diphtheriae* and NDC was investigated *in silico* by mapping the trimmed reads of NDC genomes to the plasmid assembly and visualized in IGV v2.3.61 [62].

### Virulence factor predictions for NDC isolates

Protein-coding genes were predicted from the assemblies of 56 NDC isolates (* C. pseudodiphtheriticum* =52, *C. propinquum* =4) and annotated using Prokka v 1.14.5 [63]. Orthologous gene clusters within each species were determined with Roary v 1.007002 [64] at different blastp identity levels (90 %–98 %, step-size=1 %). Due to the small sample size of *C. propinquum* isolates, two NCBI *C. propinquum* references (NCBI accession numbers: CP068160 and CP068161) were also included in the pan-genome calculation. A blastp identity threshold of 90 % was selected for downstream analysis to resolve multiple paralogues matching the virulence factor SrtC. A single sequence was randomly selected from each orthologous cluster and the concatenated protein sequences representing each species were further analysed by VFDB.

## Results

### Microbiological characterizations

Diagnostic microbiological assays identified the majority of *C. diphtheriae* case isolates (53/56) as toxigenic biovar mitis, with one isolate (PC0734) as NTTB biovar mitis, and two isolates (PC0697, PC0742) as non-toxigenic biovar gravis. Antimicrobial susceptibility testing indicated that 96.4 %(54/56) of the *C. diphtheriae* isolates exhibited intermediate resistance to penicillin according to CLSI interpretive category breakpoints. The non-toxigenic biovar gravis isolate PC0697, displayed the highest MIC for penicillin (2 µg ml^−1^) as well as resistance to erythromycin and clindamycin. In contrast, all NDC isolates were sensitive to penicillin, but 80.4 %(45/56) were not susceptible to erythromycin. Additionally, 87.5 %(49/56) of the NDC isolates were resistant to clindamycin and 8.9 %(5/56) were resistant to rifampicin, while 5.4 %(3/56) exhibited intermediate resistance to rifampicin. Altogether, 80.4 %(45/56) of the NDC isolates exhibited resistance to multiple antibiotics, which notably included erythromycin, recognized as a frontline treatment for respiratory diphtheria. Detailed microbiologic and antibiotic susceptibility results for all isolates are summarized in Table S2, where discrepant interpretations for penicillin under alternative breakpoint definitions are noted for three isolates (PC0697 [R], PC0715 [I], and PC0753 [I]).

### Genetic relatedness among *C. diphtheriae* case isolates

Both nasal and throat swabs are recommended for diagnostic confirmation of suspected diphtheria [8] and three cases yielded *C. diphthe* riae isolates from both swab sites (Table 1). Isolates from patient no. 1 (PC0691 and PC0743) differed by 1 SNP but otherwise shared the same toxigenicity, biovar, ST and antibiotic resistance profile, indicating that both swabs had sampled the same infecting population of *C. diphtheriae *. By contrast, isolate pairs from patient no. 2 (PC0715 and PC0742) and patient no. 3 (PC0697 and PC0737) differed in toxigenicity, biovar and ST, suggesting both patients were colonized by two strains of *C. diphtheriae *.

**Table 1.** Coinfection pairs of diphtheria case isolates

Patient no.	CDC ID	Collection date	Location	Swab	ST	Biovar	Elek	No. of SNPs	Macrolide resistance*
1	PC0691 PC0743	12/20/2017	Balukhali	Throat Nasal	ST453 ST453	mitis mitis	positive positive	1	negative negative
2	PC0715 PC0742	12/19/2017	Balukhali	Throat Nasal	ST835 ST123	mitis gravis	positive negative	na	negative negative
3	PC0697 PC0737 PC0738†	12/23/2017	Jamtoli	Throat Nasal Nasal	ST363 ST453	gravis mitis	negative positive	na	positive negative positive

*According to CLSI breakpoints. See Table S2 for MIC values.

†PC0738 was identified as *C. pseudodiphtheriticum* co-circulating with diphtheria case isolates in patient no. 3.

**Fig. 1.**
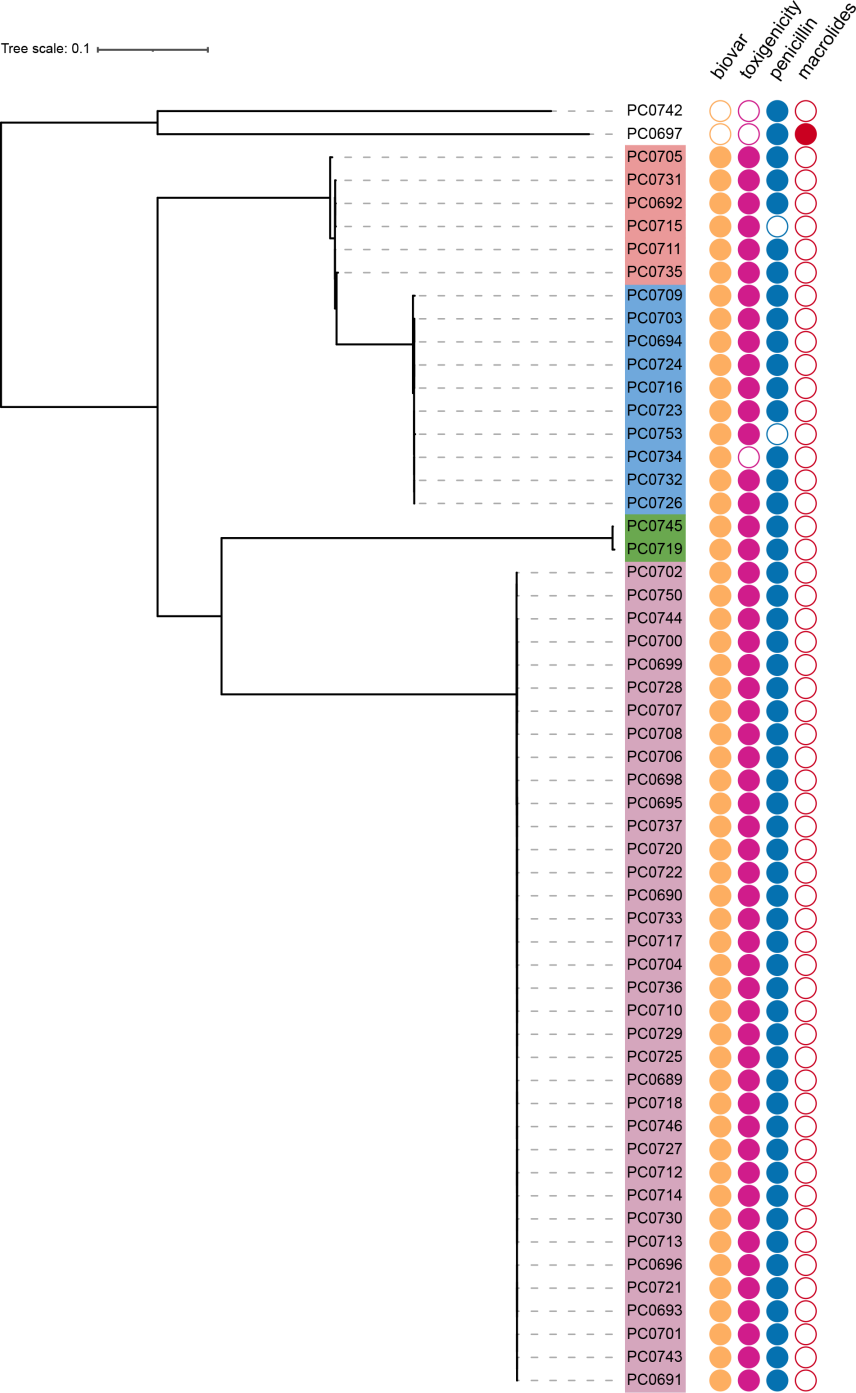
Phylogenetic reconstruction of Bangladesh *C. diphtheriae* isolates from 38 024 core variable sites using maximum likelihood. Four detected clusters are shaded in different colours. The corresponding microbiological characteristics of each isolate are indicated next to the tree. Filled circles represent biovar (mitis, yellow), toxigenicity (toxigenic, purple), susceptibility to penicillin (intermediate resistance, blue) or macrolides (resistance, red). Scale bar indicates substitutions per site.

The phylogeny of all 56 outbreak *C. diphtheriae* isolates was reconstructed from 38 024 core SNPs to investigate their genetic relatedness using the complete genome assembly of PC0696 (accession no. CP040525) as the reference (Fig. 1). The isolates grouped into four clusters of varied sizes (cluster 1 : 6 isolates; cluster 2 : 10 isolates; cluster 3 : 2 isolates; cluster 4 : 36 isolates), each separated by >15 000 SNPs, along with two sporadic isolates. Notably, both sporadic non-toxigenic isolates were recovered from cases yielding two *C. diphtheriae* isolates (patients no. 2 and no. 3) and the cocultured, toxigenic isolates from these cases were members of a phylogenetic cluster (cluster 1 or cluster 4). Replicate isolates from patient no. 1 (PC0691 and PC0743) belonged to cluster 4, while all other isolates in the four clusters each represented a unique case. Within each of the four clusters, pairwise genetic distances were less than 75 SNPs (Table 2). Additionally, MLST determined from trimmed sequencing reads identified seven unique STs among the *C. diphtheriae* isolates, each of which was associated with a discrete outbreak cluster. Only cluster 4 comprised two STs that differed by one allele, and neither ST was observed in other clusters. Three of the observed STs were novel and submitted to the Institut Pasteur database (https://bigsdb.pasteur.fr/diphtheria/) for assignment as ST835, ST836 and ST837.

**Table 2.** Four clusters were identified based on phylogeny and pairwise SNP distances

Group	No. of isolates	Sequence type (*atpA-dnaE-dnaK-fusA-leuA-odhA-rpoB*)	Within cluster pairwise SNP distance range
Cluster 1	6	ST835 (2-10-3-1-7-97-14)	0~47 SNPs
Cluster 2	10	ST836 (22-10-3-1-3-3-2)	2~42 SNPs
Cluster 3	2	ST258 (2-4-8-1-7-3-9)	70 SNPs
Cluster 4	35 1	ST453 (22-3-94-2-3-3-3) ST837 (22-3-94-47-3-3-3)	0~59 SNPs
Sporadic cases	2	ST363 (3-1-20-48-3-16-44) ST123 (2-1-45-1-5-3-9)	17 404 SNPs

**Fig. 2.**
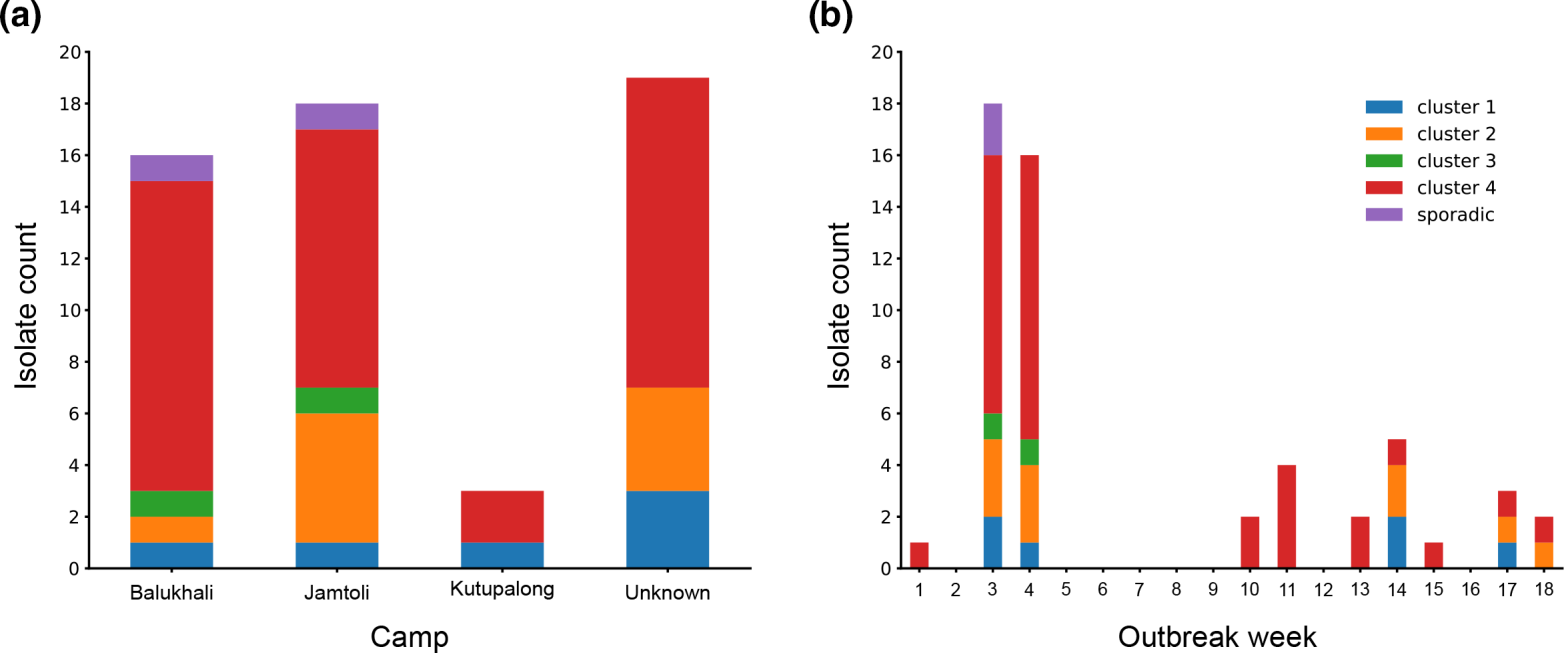
Geographical and temporal origins of *C. diphtheriae* isolates from the Bangladesh outbreak. (**a**) Geographical locations (camp) of the *C. diphtheriae* case clusters identified during the outbreak from 9 December 2017 to 7 April 2018. (**b**) Weekly identified *C. diphtheriae* clusters in Bangladesh during the same investigation period. All the collection dates have been normalized into week numbers. The 7 day intensive study period was between 19–25 December 2017 (weeks 3 and 4).

All four clusters were recovered from cases in multiple camp sites and persisted throughout the course of the 18 week outbreak period, with cluster 4 (ST453 and ST837) recovered most frequently (Fig. 2a, b). Within each cluster, phylogenetic subtrees did not reveal any overall correlation with collection date or camp site (Fig. S1). However, each cluster did include small groups of isolates differing by as few as 0 SNPs that were often, but not always, recovered from the same camp location and likely represent linked cases.

Phylogenetic placement of the Bangladesh outbreak *C. diphtheriae* isolates was further explored within the context of publicly available data within NCBI’s SRA (*n*=704) and genome database (*n*=34) meeting quality criteria described in Methods, which included reported outbreak clusters from Switzerland, Somalia and Yemen as well as other geographically defined clusters (Fig. S2). Among these, eight isolates (seven assemblies and one complete genome) from an outbreak reported in Malaysia in 2016 also exhibited ST453 and were related to isolates of cluster 4 from this study (Fig. S1C). Pairwise SNP distances between isolates from the two outbreaks were fewer than 100 SNPs, consistent with the diversity observed among the 36 Bangladesh isolates within cluster 4 (ST453 and ST837). In addition, one isolate collected in 1994 from Thailand (SRA: ERR3932547) clustered together with two Bangladesh isolates (cluster 3, ST258) with pairwise SNP distances of 505 and 510 SNPs, respectively.

### Genetic determinants of antibiotic resistance and diphtheria toxin

**Fig. 3.**
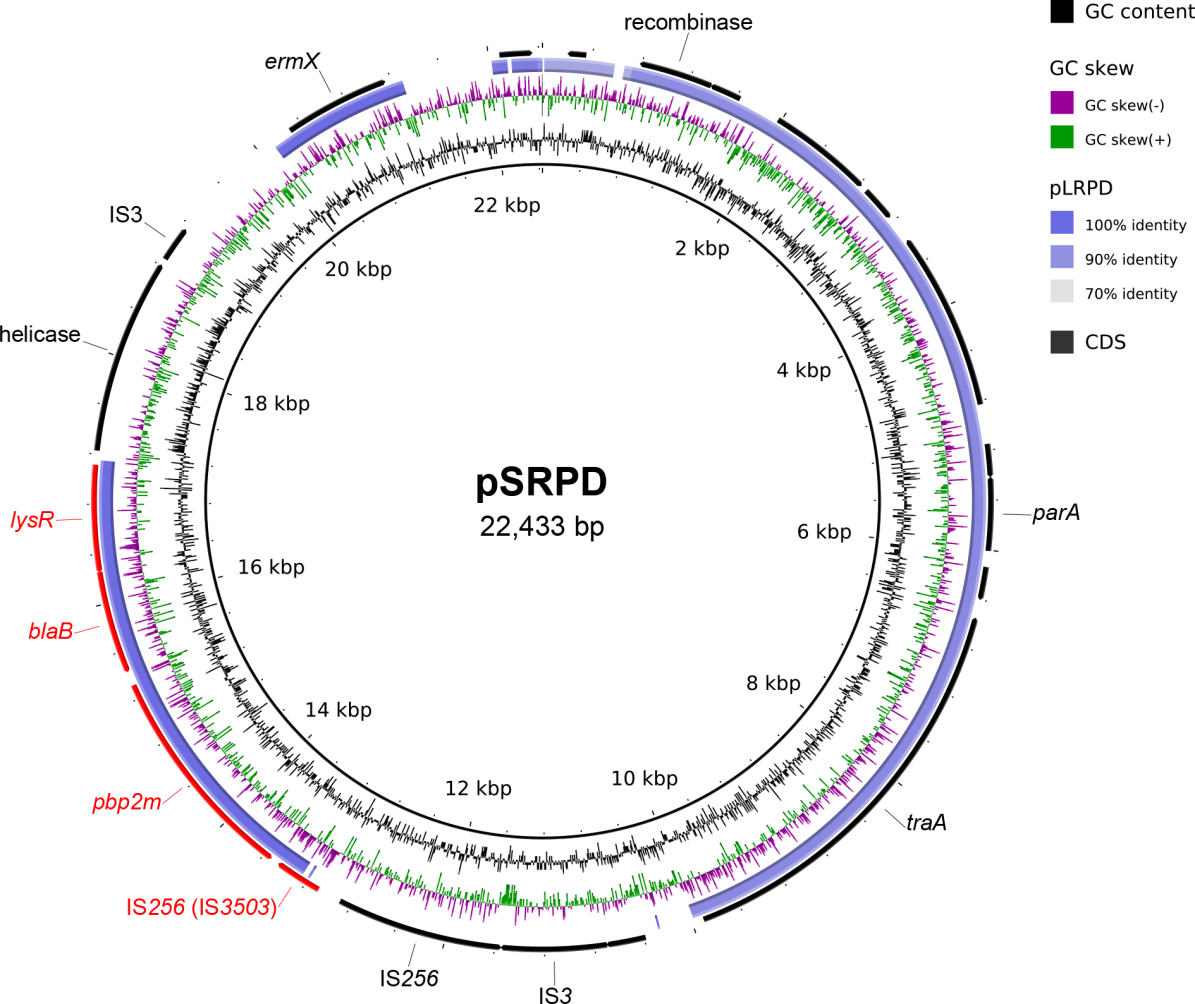
Map of novel plasmid pSRPD from the *C. diphtheriae* isolate PC0697. G+C % content is the inner black tilde; GC skew is the green/purple tildes; blue ring represents comparative plasmid analysis against reported plasmid (pLRPD). The outermost ring represents predicted CDSs, which are portrayed by arrows with important ones labelled with either gene name or predicted gene function. The CDSs in red represents the *pbp*-containing transposable unit (PCU).

Assembled genomes were investigated to detect the presence of known genetic determinants of antibiotic resistances. The majority of *C. diphtheriae* isolates (54/56) showed intermediate resistance to penicillin and all were screened for the presence of eight previously reported penicillin-binding protein (PBP) sequences [21]. Isolates were stratified based on penicillin MICs and compared according to their complement of detected *pbp* genes (Table S3). Although no clear association between penicillin MIC and the number of encoded *pbp* genes was observed, PC0697 exhibited the highest penicillin MIC and was the only isolate to harbour *pbp2m*, which has been reported to confer elevated penicillin resistance [21]. Notably, PC0697 was resistant to erythromycin, and had the similar MIC for azithromycin and clarithromycin. Since it was resistant to erythromycin, it was likely resistant to these two antibiotics though no breakpoints have been established for them (Table S1, Table S2). The common genetic determinant for this phenotype, *ermX*, was similarly detected in the genome of PC0697 and matched with 100 % identity to that in plasmid pNG2 (accession no. NC_005001.1). To define the genomic context of both resistance genes, PC0697 was sequenced further using a long-read platform. *De novo* assembly revealed the presence of a putative 22,433 bp plasmid that included both the *pbp2m* and *ermX* genes, which we propose to name as pSRPD (for small resistance plasmid of *C. diphtheriae *) (Fig. 3). Further annotation of pSRPD identified a transposable PBP-containing unit (PCU) comprising a gene cassette of *pbp2m*, *blaB*, *lysR* and truncated IS3503, which shared 99.8 % sequence identity to the PCU in pLRPD and was similar in organization to previously described group 1 PCUs [21]. Plasmid annotation also revealed replication machinery (replication initiation protein, *parA* and helicase) and conjugation machinery (*traA* and recombinase) that together were >91.8 % identical to pLRPD as well as multiple insertion sequence (IS) elements (IS*256* and IS*3* superfamily).

Additionally, our genetic analysis confirmed the presence of intact diphtheria toxin gene in the 53 toxigenic *C. diphtheriae* isolates, and the absence in two non-toxigenic isolates (PC0697 and PC0742). While the genome of PC0742 included no detectable corynephage fragments, PC0697 did possess remnants of sequence homology to the corynephage in reference NCTC13129 (accession no. NC_002935) but not the toxin gene. The only NTTB isolate (PC0734) had a single nucleotide deletion at position 488 of the diphtheria toxin gene, leading to a frameshift, which was distinct from NTTB corynephage types described elsewhere [65].

### Characterization of cocirculating NDC isolates

**Fig. 4.**
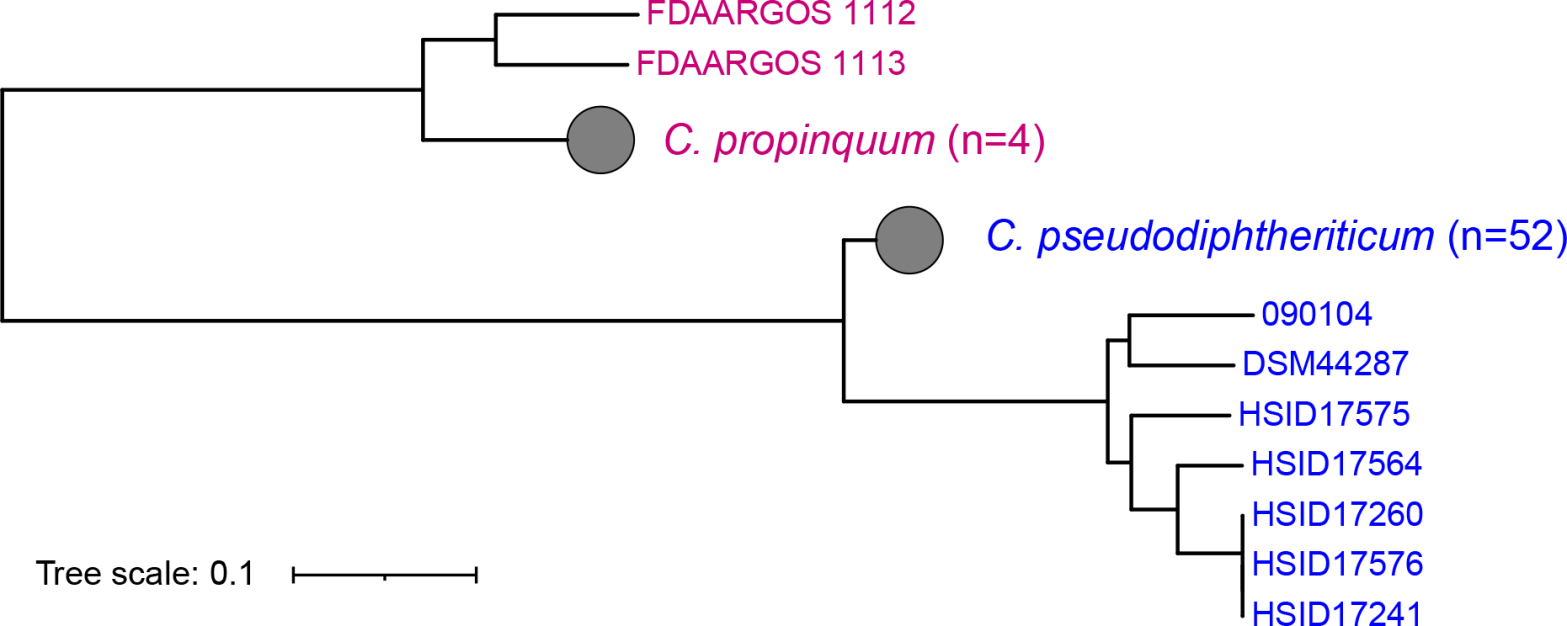
Phylogeny reconstructions for NDC isolates from 31 946 core variable sites using maximum likelihood. Isolates belonging to *C. pseudodiphtheriticum* were highlighted in blue while *C. propinquum* in purple. Grey circles represent collapsed clades of Bangladesh NDC isolates from the two species. Scale bar indicates substitutions per site.

Laboratory culture during outbreak investigation revealed cocirculating NDC species *C. pseudodiphtheriticum* [8] and 56 isolates were subjected to WGS for further characterization. Four isolates (PC1113, PC1129, PC1151 and PC1142) were most similar to *C. propinquum* with an average ANI of 97.4 % compared to two available references (accession no. CP068160 and CP068161), and an average ANI of 88.8 % compared to a *C. pseudodiphtheriticum* reference (accession no. CP091864). Accordingly, phylogenetic reconstruction of the 56 NDC isolates with nine publicly available genomes delineated the two species between two clear branches separated by approximately 95 000 SNPs (Fig. 4). Subtrees calculated for each species indicated that NDC isolates recovered during the outbreak were diverse, differing by an average 25 561 and 6963 pairwise SNPs for *C. propinquum* and *C. pseudodiphtheriticum *, respectively (Fig. S3). Each were also distinct from public references, with *C. propinquum* separated by at least 34 138 SNPs and *C. pseudodiphtheriticum* separated by at least 26 474 SNPs.

Most NDC isolates in this study were not susceptible to erythromycin and *ermX* was detected with the raw genomic sequencing read data. Further query of *de novo* assemblies to investigate the genetic context revealed that *ermX* appeared in contigs also encoding IS elements. Similarly, the four closed genomes of NDC isolates [42] indicated that *ermX* was encoded within the chromosome, rather than plasmids, with sequencing coverage depth comparable to that of the entire genome, where it was also flanked by numerous IS elements. Multiple sequence alignment of the adjacent regions around *ermX* in these closed genomes further revealed that overall placement within the chromosome was variable (Fig. S4), suggesting potential acquisition through horizontal transfer. The same was true for *C. pseudodiphtheriticum* PC0738, despite being cocultured from patient no. 3 with *C. diphtheria* PC0697, which harboured *ermX*-containing pSRPD. To better understand the ecology of these two NDC species, gene content of the 56 isolates was explored and potential virulence factors were annotated using VFDB. The two species of *C. propinquum* and *C. pseudodiphthericum* shared very similar virulence factor profiles (Table S4 and Table S5), which were quite different from the virulence factor complement of the well-characterized *C. diphtheriae* reference NCTC13129 (accession no. NC_002935). For example, NDC isolates encoded iron uptake systems such as ABC-transporters and ABC-haem transporter similar to NCTC13129 but lacked the majority of adherence factors including *spaA-, spaD-* and *spaH-*type pili. However, genomes of both *C. propinquum* and *C. pseudodiphthericum* were predicted to harbour additional virulence factors common to pathogens such as *Helicobacter *, *Mycobacterium* and *Klebsiella *, with predicted functions like acid resistance, antiphagocytosis and copper uptake.

## Discussion

Between 2017 and 2019, a large diphtheria outbreak was reported within camps of forcibly displaced Myanmar nationals residing in Bangladesh [9, 66, 67]; laboratory and epidemiological investigation of which yielded cultured bacterial isolates for further study [8]. These isolates were recovered from cases during the first 18 weeks of the outbreak, which included the period of peak incidence in the epidemic curve [9]. Here, we have supplemented standard microbiological characterization with WGS and bioinformatics to shine light on the genomic characteristics of *C. diphtheriae* and cocirculating NDC species isolated from a subset of cases in the outbreak.

Previous genomic analyses of diphtheria outbreak investigations have shown that isolates from cases infected through a common transmission network often form a single cluster with fewer than 150 pairwise SNP differences and fewer than 40 SNP differences often separate isolates from epidemiologically linked cases, while unlinked sporadic isolates may differ by over 10 000 SNPs [16, 22, 24]. Consistent with this SNP threshold, the results here suggested that at least four distinct phylogenetic clusters, not the clonal spread of a single cluster, contributed to the resurgence of diphtheria among the refugee population residing in Bangladesh. Each cluster included smaller groups of very similar isolates indicative of recent transmission among potentially linked cases. While most of these groups were limited to one camp, some included isolates from two camps or, more frequently, unknown locations. Case ascertainment and data collection were challenging due to difficulty with patient follow up as well as language barriers between healthcare workers and patients. Nevertheless, the available data suggested that the four predominant outbreak clusters likely spanned multiple refugee camps and persisted over the entire course of the 18 week outbreak investigation. Additional global phylogenetic analyses including publicly available sequence data identified previously reported *C. diphtheriae* isolates from other Asian countries such as Thailand and Malaysia [14, 15, 68] were closely related with two clusters (cluster3: ST258; cluster 4: ST453, ST837). Taken together, these observations are consistent with other large, poly-clonal diphtheria outbreaks recently reported [7, 23, 69], reflecting the public health crises that can emerge as almost a million people fleeing violence gathered in densely populated refugee camps with limited health services.

Isolates of *C. diphtheriae* recovered from outbreak cases were overwhelmingly characterized as toxigenic biovar mitis (53/56, 94.6 %), confirming that diphtheria toxin remains an important virulence factor to the spread of disease. Notably, the two non-toxigenic biovar gravis isolates were recovered from cases also infected by a toxigenic biovar mitis isolate and thus provided direct evidence of mixing between toxigenic and non-toxigenic *C. diphtheriae* within the same host patient. Antibiotic susceptibility profiling indicated only intermediate penicillin resistance was common among toxigenic and non-toxigenic *C. diphtheriae* isolates, confirming that preferred antibiotics for treatment (penicillin and erythromycin) were still active against *C. diphtheriae* isolates circulating in this outbreak. However, one non-toxigenic *C. diphtheriae* isolate (PC0697) exhibited multidrug resistance to both first-line antibiotics, and genetic determinants (*pbp2m*, *ermX*) were detected in a putative plasmid by WGS. This non-toxigenic isolate harbouring a multidrug resistance plasmid was recovered from a patient also infected by toxigenic *C. diphtheriae *, which raises questions regarding the potential for horizontal transfer of the eponymous corynephage, plasmids or both among coexisting genotypes [70, 71]. Although transmissibility of this plasmid among *C. diphtheriae* strains requires further investigation, reports elsewhere of plasmid encoded *pbp2m* and *ermX* together [21], as well as varied *ermX* [72] and integrons [73], highlight the public health threat of mobile element driven spread of antibiotic resistance in *C. diphtheriae *.

Previous epidemiologic and laboratory investigation of this outbreak also recovered 56 NDC isolates, providing a unique opportunity for genomic analyses of these under-characterized species. NDC are considered commensal members of the normal human microflora but have gradually been recognized as emerging opportunistic pathogens [74]. Their recovery during this outbreak may simply have resulted from the intense investigation and growth on agar media used to culture *C. diphtheriae *, not because they caused diphtheria-like illnesses. However, most NDC isolates in this study were resistant to macrolides and the known resistance determinant *ermX* was detected in the WGS data, which suggested placement within the chromosome and flanked with IS elements. No genomic evidence for cross-species exchange of an *ermX*-encoding plasmid was observed in a patient coinfected with *C. diphtheriae* and NDC. Therefore, widespread utilization of macrolides, specifically azithromycin, to control the diphtheria outbreak [8, 9] may in turn have facilitated the growth and spread of NDC in this population.

Comparative genomics correctly identified 4/56 isolates as *C. propinquum *, all of which were previously described as *C. pseudodiphtheriticum* according to Weil *et al*. [8]. Methods for microbiological (e.g. API Coryne strip) or molecular identification (e.g. 16S rRNA gene sequencing, RT-PCR) of NDC cannot adequately differentiate these two species, highlighting the importance of combining multiple assays including matrix-assisted laser desorption/ionization-time of flight (MALDI-TOF), *rpoB* PCR, and genomics to correctly identify NDC species, which share very similar biochemical profiles [75]. Public databases include few sequenced representatives of either species. Our preliminary investigation of their virulence factor gene contents here confirmed that isolates of both species lacked most virulence determinants present in *C. diphtheriae *. Furthermore, phylogenetic reconstruction within each species indicated that the NDC isolates recovered from this outbreak were genetically diverse, and thus more likely represent commensal microflora rather than outbreak transmission between individuals.

The present work provides added depth for understanding characteristics of strains circulating in this outbreak, their relatedness to strains common in neighbouring regions, and their antibiotic resistance patterns. Notably, our evaluation indicates that disease in this outbreak resulted from concurrent transmission of multiple distinct toxigenic *C. diphtheriae* strains, distinguishable by discrete STs and SNP distances. While genomic data are not available in real-time during an outbreak and thus unable to inform immediate public health action, they may reinforce ongoing and future public health interventions. Our data confirm that toxigenic *C. diphtheriae* strains circulate not only in this outbreak but also regionally, reiterating the importance of routine diphtheria toxoid vaccination on a broad scale. Additionally, these data highlight possible future resistance to standard antibiotic treatment, underscoring the need for continued surveillance for diphtheria and testing for antimicrobial resistance to potentially inform recommendations regarding antibiotic treatment during future regional outbreaks. All of which demonstrate the critical and actionable information that WGS can provide to public health.

## Supplementary Data

Supplementary material 1

Supplementary material 2

Supplementary material 3

Supplementary material 4
