# Supplementary material for: Genomic characterization of cocirculating Corynebacterium diphtheriae and non-diphtheritic Corynebacterium species among forcibly displaced Myanmar nationals, 2017–2019
Source: Microb Genom. 2023 Sep 15;9(9):001085. doi: 10.1099/mgen.0.001085 (PMC10569726; doi:10.1099/mgen.0.001085)
Supplement: Supplementary material 1 [file mgen-9-1085-s001.pdf]

Table S1: Antibiotic minimum inhibitory concentrations (µg/mL) breakpoints and interpretive categories for Corynebacterium spp. as defined by the Clinical and Laboratory Standards Institute (CLSI)*.

Antibiotic	Susceptible [S]	Intermediate [I]	Resistant [R]
Penicillin	≤0.12	0.25-2	≥4
Amoxicillin	NA	NA	NA
Meropenem	≤0.25	0.5	≥1
Vancomycin	≤2	NA	NA
Daptomycin	≤1	NA	NA
Azithromycin	NA	NA	NA
Erythromycin	≤0.5	1	≥2
Clarithromycin	NA	NA	NA
Levofloxacin	NA	NA	NA
Clindamycin	≤0.5	1-2	≥4
Rifampicin	≤1	2	≥4

NA: no interpretive cutoff defined

*The European Committee on Antimicrobial Susceptibility Testing (EUCAST) defines alternate breakpoints for penicillin, susceptible: ≤0.001, intermediate: 0.001-1, resistant: >1; and macrolides, susceptible: ≤0.06, resistant: >0.06 (https://www.eucast.org/).

Table S2: Summary table for metadata, microbiological identifications and sequence accession numbers for Bangladesh *C. diphtheriae* and NDC isolates – *Xiaoli_TableS2.xlsx*

Table S3: Genetic determinant screening of penicillin resistances for representative case isolates

CDC ID	Species	MIC (µg/mL)	Interpretation	Presence of *pbp* (>97% identity to amino acid references)
PC0697	*C. diphtheriae*	1.5	Intermediate	*pbp1a, 1b,2a,2b,2c, 4, 4b,* ***2m***
PC0696	*C. diphtheriae*	0.5	Intermediate	*pbp1a, 1b,2a,2b,2c,4, 4b*
PC0685	*C. diphtheriae*	0.38	Intermediate	*pbp1a, 1b,2a,2b,2c,4, 4b*
PC0753	*C. diphtheriae*	0.125	Sensitive	*pbp1a, 1b,2a,2b,2c,4, 4b*

Table S4: Virulence factor predictions for co-circulating *C. propinquum* – *Xiaoli_TableS4.xlsx*

Table S5: Virulence factor predictions for co-circulating *C. pseudodiphtheriticum* – *Xiaoli_TableS5.xlsx*

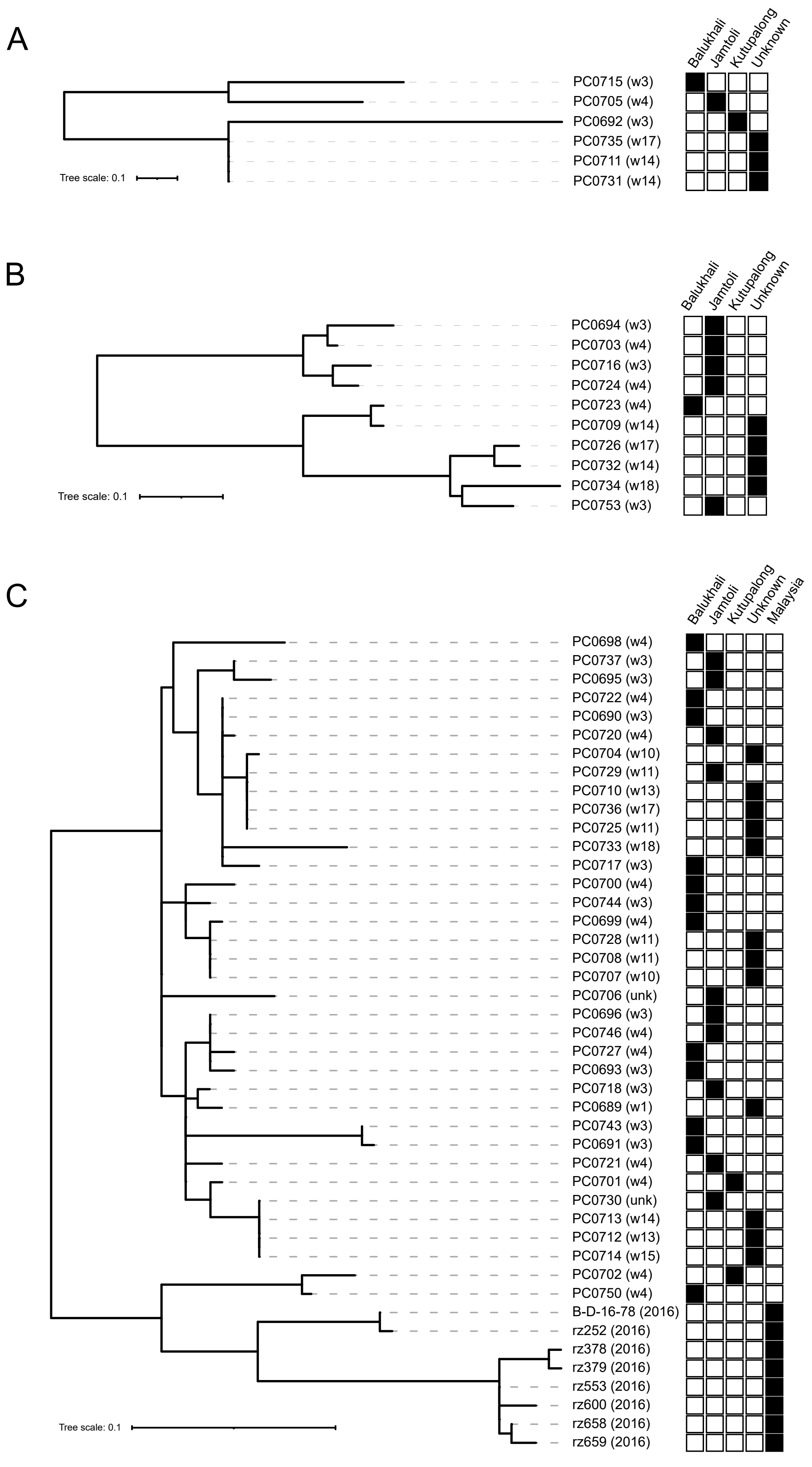

Figure S1: Phylogenetic reconstruction of Bangladesh *C. diphtheriae* isolates from (a) cluster 1, (b) cluster 2, and (c) cluster 4 using maximum likelihood. The number of core variable sites in each cluster were 62, 69, and 170 bp, respectively. Geographic and temporal information for each isolate are indicated next to the tree. Collection dates were normalized to outbreak week number for Bangladesh isolates and year for Malaysian isolates, as indicated in parentheses. A group of eight *C. diphtheriae* isolates from 2016 and Malaysia share the same ST with cluster 4 (c). Scale bars indicate substitutions per site.

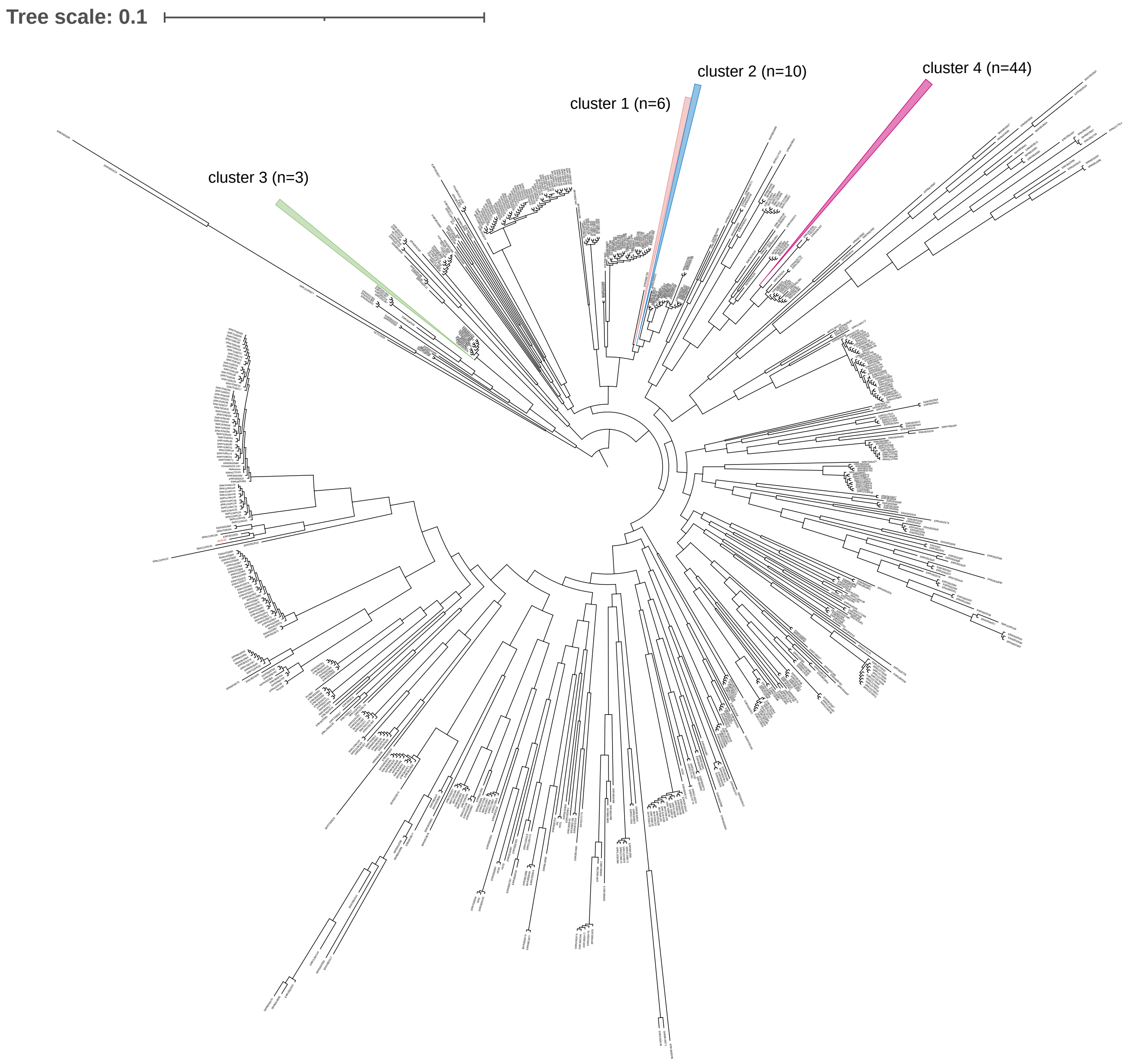

Figure S2: Phylogenetic reconstruction of publicly available *C. diphtheriae* genomes with Bangladesh isolate collection from 138,694 core variable sites using maximum likelihood. Isolates including those collapsed with Bangladesh ones belonging to previously defined four clusters were highlighted in different colors (cluster1, pink; cluster2, blue; cluster3, green; cluster4, purple). Cluster 3 included one Thailand genome, and cluster 4 contained eight Malaysian genomes. Scale bar indicates substitutions per site.

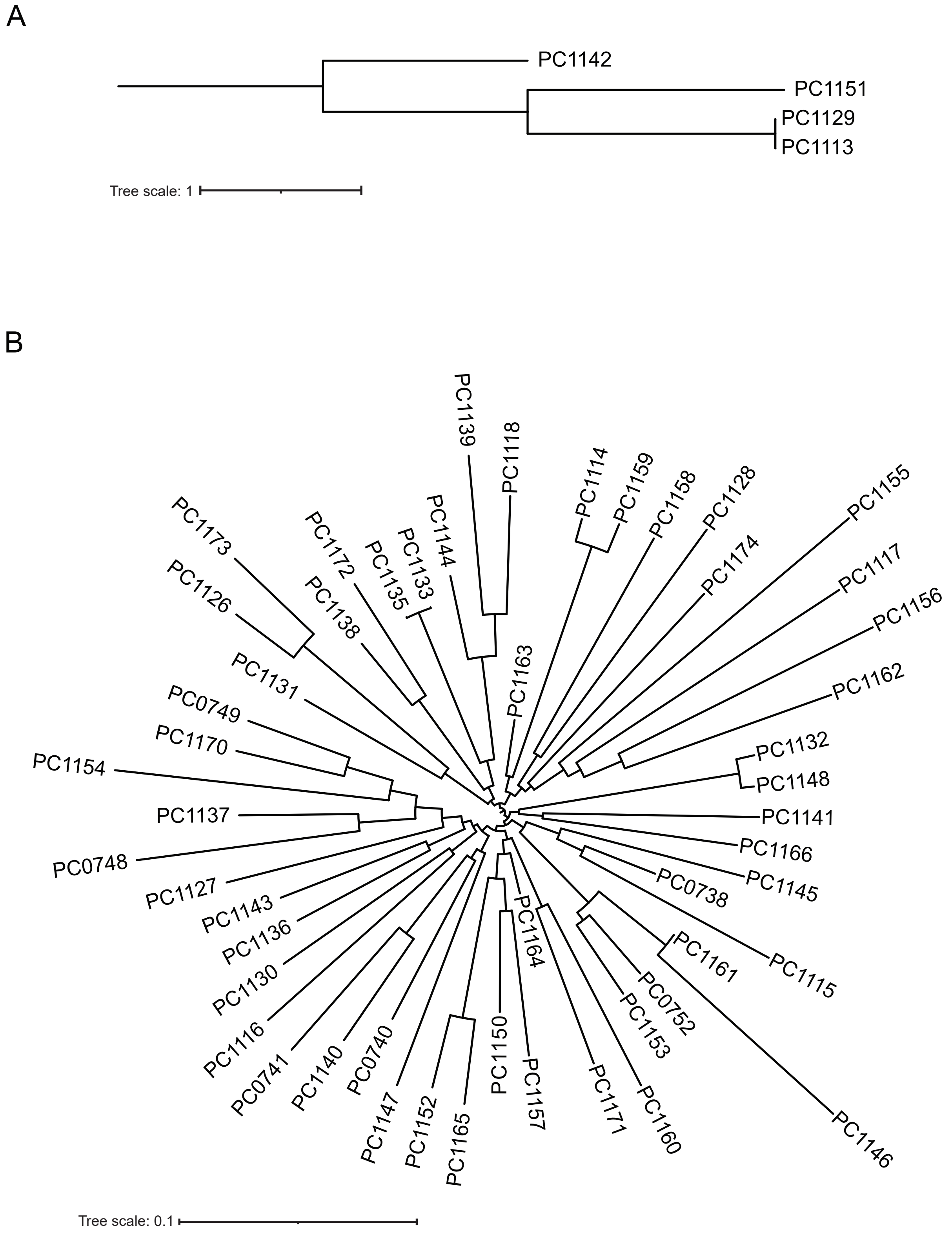

Figure S3: Phylogenetic reconstruction of Bangladesh NDC isolates of (a) *C. propinquum*, and (b) *C. pseudodiphtheriticum* using maximum likelihood. The number of core variable sites in each were 46,517 and 61,984 bp, respectively. Scale bars indicate substitutions per site.

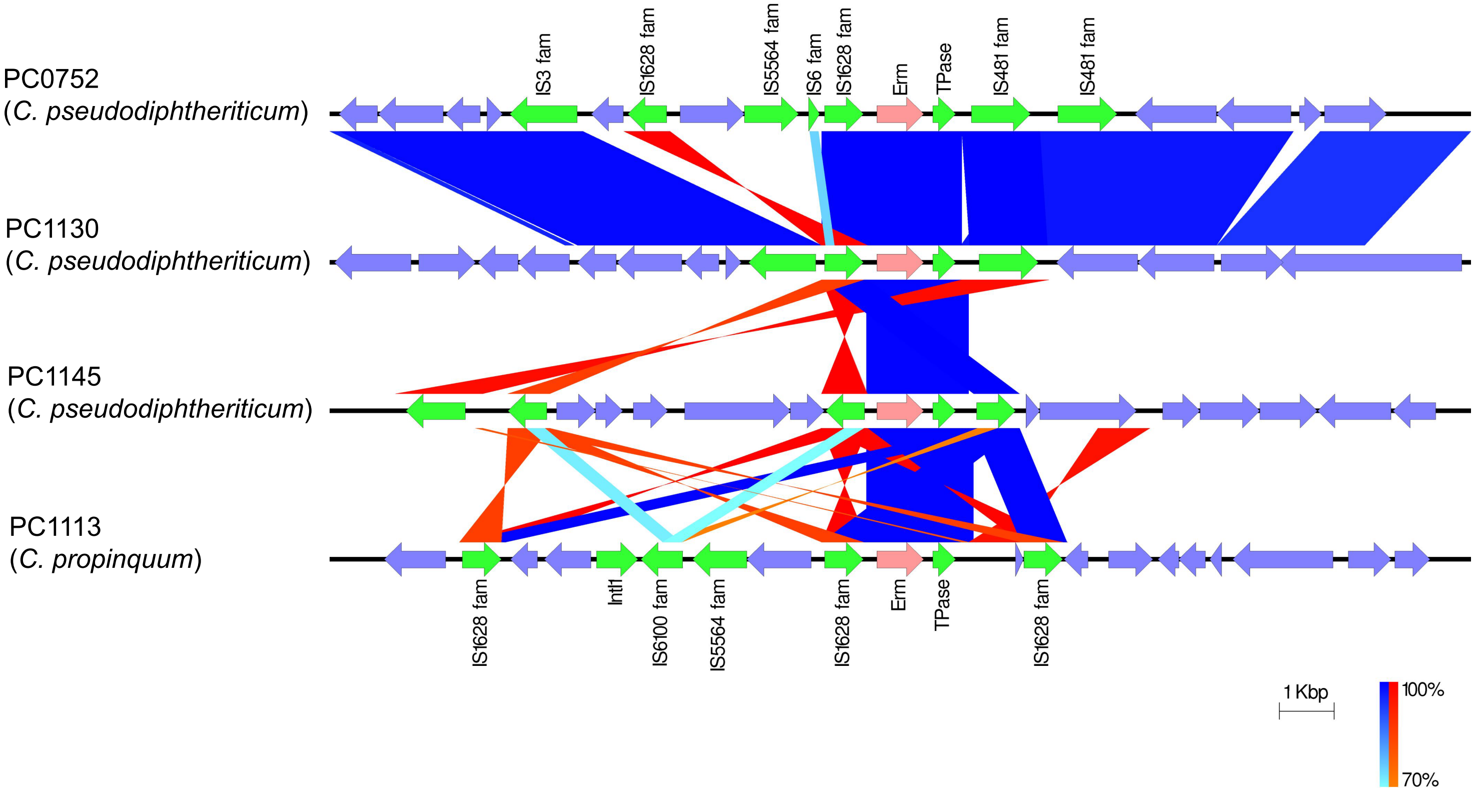

Figure S4: Multiple sequence alignment of *ermX* flanking regions in closed genome assemblies for four NDC isolates (PC0752, PC1130, PC1145, and PC1113). BLASTn comparisons of adjacent regions (10kb) around *ermX* and were visualized using Easyfig. Blue/light blue shaded regions connect homologous sequences with either high or low identity (max=100%, min=70%). Red/orange shaded regions connect reverse complemented homologous sequences with either high or low identity (max=100%, min=70%). Purple arrows represent CDSs, while pink and green arrows designate the location of ermX and IS-elements related CDSs, respectively. TPase stands for transposase, IS fam for IS family transposase, and IntI stands for class 1 integron-integrase.
